# Morphological and molecular changes in the murine placenta exposed to normobaric hypoxia throughout pregnancy

**DOI:** 10.1113/JP271073

**Published:** 2015-09-15

**Authors:** Hannah Matheson, Jan H. W. Veerbeek, D. Stephen Charnock‐Jones, Graham J. Burton, Hong Wa Yung

**Affiliations:** ^1^ Centre for Trophoblast Research, Department of Physiology, Development and NeuroscienceUniversity of CambridgeCambridgeUK; ^2^ Department of Obstetrics and GynaecologyUniversity of Cambridge, The Rosie HospitalCambridgeUK; ^3^ National Institute for Health ResearchCambridge Comprehensive Biomedical Research CentreCambridgeUK; ^4^ Birth Center and the Division of Woman and BabyUniversity Medical Center UtrechtUtrechtThe Netherlands

## Abstract

**Key points:**

- Exposure of pregnant mice to chronic hypoxia at 13% O_2_ induces fetal growth restriction but increases placental weight.
- Sex dimorphism induces differential responses in placental weight to hypoxia. The male placenta is heavier than the female and is associated with less severe fetal growth restriction.
- Increases in maternal arterial/venous blood spaces and higher protein kinase B (Akt)‐mechanistic target of rapamycin growth signalling could contribute to the heavier hypoxic placenta.
- Placental endoplasmic reticulum stress is elevated equally in both sexes in response to hypoxia. In comparison, oxidative stress is only apparent in female placentas.
- Chronic hypoxia induces down‐regulation of placental mitochondrial electron transport chain complexes protein subunits but does not cause intracellular energy depletion.

**Abstract:**

Chronic hypoxia is a common complication of pregnancy, arising through malperfusion of the placenta or pregnancy at high altitude. The present study investigated the effects of hypoxia on the growth of the placenta, which is the organ that interfaces between the mother and her fetus. Mice were housed in an hypoxic environment for the whole of gestation. An atmosphere of 13% oxygen induced fetal growth restriction (1182 ± 9 mg, *n* = 90 *vs*. 1044 ± 11 mg, *n* = 62, *P* < 0.05) but enhanced placental weight (907 ± 11 mg, *n* = 90 *vs*. 998 ± 15 mg, *n* = 62,*P* < 0.05). Stereological analyses revealed an increase in the volume of maternal blood spaces in the placenta, consistent with increased flow. At the molecular level, we observed activation of the protein kinase B (Akt)‐mechanistic target of rapamycin growth and proliferation pathway. Chronic hypoxia also triggered mild endoplasmic reticulum stress, a conserved homeostatic response that mediates translational arrest through phosphorylation of eukaryotic initiation factor 2 subunit α. Surprisingly, although subunits of the mitochondrial electron transport chain complexes were reduced at the protein level, there was no evidence of intracellular energy depletion. Finally, we demonstrated sex‐specific placental responses to chronic hypoxia. Placentas from male fetuses were heavier (1082 ± 2 mg, *n* = 30 *vs*. 928 ± 2 mg, *n* = 34, *P* < 0.05) and less susceptible to hypoxia‐induced oxidative stress than those from females. Their capacity to adapt may explain why male fetuses were significantly less growth restricted at embryonic day 18.5 than their female counterparts. These findings are consistent with the concept that male fetuses are more aggressive with respect to their nutrient demands, which may place them at greater risk of adverse outcomes under limiting conditions.

## Key points

- Exposure of pregnant mice to chronic hypoxia at 13% O_2_ induces fetal growth restriction but increases placental weight.
- Sex dimorphism induces differential responses in placental weight to hypoxia. The male placenta is heavier than the female and is associated with less severe fetal growth restriction.
- Increases in maternal arterial/venous blood spaces and higher protein kinase B (Akt)‐mechanistic target of rapamycin growth signalling could contribute to the heavier hypoxic placenta.
- Placental endoplasmic reticulum stress is elevated equally in both sexes in response to hypoxia. In comparison, oxidative stress is only apparent in female placentas.
- Chronic hypoxia induces down‐regulation of placental mitochondrial electron transport chain complexes protein subunits but does not cause intracellular energy depletion.

**Abbreviations**
Akt‐mTOR: protein kinase B‐mechanistic target of rapamycin
AMPK: AMP‐activating kinase
E: embryonic day
4EBP‐1: 4E binding protein 1
ER: endoplasmic reticulum
eIF2α: eukaryotic initiation factor 2 subunit α
eIF4: eukaryotic initiation factor 4
ETC: electron transport chain
HSP: heat shock protein
IL: interleukin
IRE1: inositol requiring endoribonuclease 1
PERK: protein kinase RNA‐like endoplasmic reticulum kinase
TNF: tumour necrosis factor
UPR: unfolded protein response
XBP‐1: X‐box binding protein 1

## Introduction

Sir Joseph Barcroft asserted that the fetus develops under an oxygen tension comparable to that experienced on the summit of Mount Everest; the ‘Everest *in utero*’ hypothesis (Barcroft, 1946). The experiments he performed, and those of others, demonstrated clearly that oxygen availability is an important determinant of fetal growth. The question arises as to what happens during pregnancy when the feto‐placental unit suffers hypoxia and fetal oxygenation might be expected to be reduced further. Is the placenta able to adapt to maintain the oxygen flux to the fetus? Placental hypoxia has been implicated in the pathophysiology of various complications of pregnancy, including non‐genetic or infectious causes of growth restriction, allthough it has never been confirmed by measurements performed *in vivo*. By contrast, pregnancy at altitude represents an experiment of nature in which the maternal arterial oxygen tension (*P*_a_O_2_.) is known to be significantly lowered, dropping from 95 mmHg at sea level to ∼50 mmHg at 3500–4000 m (Krampl *et al*. 2000; Postigo *et al*. 2009). This decrease is partially compensated for in terms of oxygen carriage by physiological responses, with an increase in the maternal haematocrit and haemoglobin concentration (Julian *et al*. 2009; Postigo *et al*. 2009). Nonetheless, the incidence of small‐for‐gestational age births increases in humans at high altitude, with birth weight being reduced by ∼100 g for every 1000 m of elevation (Giussani *et al*. 2001). The effect is greatest in recent migrants of non‐indigenous origin (Moore *et al*. 2001; Julian *et al*. 2007) and is independent of other risk factors, including smoking and socioeconomic class (Giussani *et al*. 2001).

In comparison with the well‐established change in birth weight in high‐altitude pregnancies, the effect on the placenta is contentious. Increases, decreases or no difference in human placental weight have all been reported (Jackson *et al*. 1987; Reshetnikova *et al*. 1994; Tissot van Patot *et al*. 2003; Zamudio, 2003; Tissot van Patot *et al*. 2010). This variability in the data is probably a result of intrinsic differences among the populations studied, such as ethnic background, dietary intake and altitudinal levels, as well as the mode of delivery and processing of the placenta. Animal studies have produced equally variable results. There was no change in placental weight when guinea‐pigs and mice were exposed to 12% oxygen throughout pregnancy (Bacon *et al*. 1984) or from embryonic day (E)14.5 (Cuffe *et al*. 2014), respectively, whereas placental weight increased in rats exposed to 13% oxygen from E6.5 onwards (Richter *et al*. 2012).

Upon adverse conditions, the fate of cells is determined by the balance between activation of stress‐response and growth signalling pathways. Hypoxia leads to an immediate reduction in energy‐demanding cellular functions aiming to conserve resources. These functions include the synthesis of non‐essential proteins, which is achieved by a selective arrest of translation mediated principally through phosphorylation of eukaryotic initiation factor eukaryotic initiation factor 2 subunit α (eIF2α) (Koritzinsky *et al*. 2006; Fahling, 2009). Over the longer term, changes in protein kinase B‐mechanistic target of rapamycin (Akt‐mTOR) signalling affect metabolic, angiogenic, anti‐oxidative and other adaptive responses that promote the survival of a cell within its new environment (Manning & Cantley, 2007; Sengupta *et al*. 2010).

The activities of many components in the Akt‐mTOR pathway are regulated by oxidative or endoplasmic reticulum (ER) stress at multiple levels, including gene expression, protein translation and phosphorylation (Koritzinsky *et al*. 2006; Yung *et al*. 2007; Yamaguchi *et al*. 2008; Yung *et al*. 2011). Phosphorylation of residues Thr308 and Ser473 critically regulates Akt activity and downstream substrate specificity (Guertin *et al*. 2006; Jacinto *et al*. 2006), whereas the phosphorylation level of 4E binding protein 1 (4EBP‐1) is the readout of mTORC1 activity and regulates protein synthesis (Hay & Sonenberg, 2004). ER stress can attenuate Akt protein translation (Yung *et al*. 2007), as well as up‐regulate gene expression of 4EBP‐1. Mice with a transgenic deletion of *Akt1* show a decrease in mTOR signalling, and a reduction in placental and fetal weight by 50% and 20%, respectively (Yang *et al*. 2003; Yung *et al*. 2008). Akt‐mTOR signalling is also reduced in human growth restricted placentas (Yung *et al*. 2008), indicating the importance of Akt‐mTOR signalling in placental growth. In a recent study, we also reported that Akt‐mTOR signalling is reduced in the human placenta from high altitude, despite no change in placental weight (Yung *et al*. 2012).

Both elevated and reduced oxidative stress have been reported in placentas from high altitude (Jefferson *et al*. 2004; Zamudio *et al*. 2007; Yung *et al*. 2012). Oxidative stress and ER stress are closely interlinked and are increased in human placentas from cases of fetal growth restriction (Burton & Yung, 2011). ER stress activates the unfolded protein response (UPR), a cellular homeostatic response to stimuli/stresses aimed at restoration of normal ER function (Walter & Ron, 2011). It comprises three evolutionarily conserved signalling pathways, including protein kinase RNA‐like endoplasmic reticulum kinase (PERK)/eIF2α, activating transcription factor 6 and inositol requiring endoribonuclease 1 (IRE1)/X‐box binding protein 1 (XBP‐1). These pathways are activated sequentially in response to ER stress, resulting in the initiation of a series of adaptive responses, including attenuation of non‐essential protein translation, an increase of ER folding capacity and facilitation of degradation of misfolded proteins.

To investigate the effects of chronic hypoxia on placental development, pregnant mice were housed from day 0.5 of gestation inside an hypoxic chamber with a normobaric atmospheric oxygen concentration of either 16% or 13%, which is equivalent to the hypobaric oxygen concentration at ∼2100 m and 3600 m, respectively. Placentas were assessed morphologically, as well as at the molecular level for adaptive responses.

## Methods

All chemicals were purchased from either Sigma‐Aldrich (Poole, UK) or Fisher Scientific UK Ltd (Loughborough, UK), except where statedotherwise. Antibodies against phospho‐Akt (Ser473), phospho‐Akt (Thr308), Akt, phospho‐4EBP‐1 (Thr37/46), 4EBP‐1, phospho‐eIF2α (Ser51), eIF2α, phospho‐p38 MAPK (Thr180/Tyr182), p38 kinase, phospho‐AMP‐activating kinase(AMPK)α(Thr172), AMPKα, phospho‐heat shock protein (HSP)27 (Ser82) and HSP27 were obtained from Cell Signalling Technology (NEB, Hitchin, UK). Antibodies against XBP‐1, GRP94 and HSP60 were obtained from Abcam (Cambridge, UK), and HSP90 and HSP70 were obtained from Enzo Life Science (Exeter, UK). Anti‐GRP78 was from Transduction Laboratories (BD Biosciences, Oxford, UK) and anti‐β‐actin was obtained from Sigma‐Aldrich. OxPhos Complex Kit for mitochondrial electron transport chain (ETC) subunits was obtained from Invitrogen (Renfrew, UK).

### Animal housing and hypoxic chamber

All experimental procedures were carried out in accordance with the UKHome Office Animals (Scientific Procedures) Act 1986 as described by Drummond (2009), which mandates ethical review. C57BL/6 mice were bred and housed in conventional cages under standard conditions (12 : 12 hlight/dark cycle at 23 ± 1°C and 60% humidity) with free access to food and water. For the experiments, virgin females (8–12 weeks of age) were paired with fertile males (minimum 10 weeks of age). The presence of a copulatory plug was considered as day 0.5 of pregnancy (term ∼20 days). The pregnant mice were randomly allocated to normoxic (20% O_2_), mild or more severe hypoxic (16% O_2_ or 13% O_2_) groups. The value of 13% O_2_ was chosen because previous studies in the rat showed that this is the lowest level that does not induce any change in food intake (Richter *et al*. 2012). Females assigned to the hypoxic groups were placed inside an hypoxic chamber, which combined a PVC isolator (PFI Plastics Ltd, Keynes, UK) with a nitrogen generator (N2MID60; Dominick Hunter Ltd, Warwick, UK), with an oxygen concentration setting of either 16% or 13%. The normoxic group was housed in the same room. Daily food and water intake in the groups were measured. Animals were killed by cervical dislocation at E18.5. Uteri were removed and immediately immersed in ice‐cold PBS. The number of pups and resorptions in each horn was counted. The placentas and fetuses were weighed, and the placentas were cut equally into two portions; one half was snap‐frozen in liquid nitrogen for western blotting analysis and the other was fixed in ice‐cold 4% paraformaldehyde in PBS for subsequent embedding in paraffin wax for stereology and immunohistochemistry. Tail tips were collected for sex determination by PCR.

### Western blotting

Western blotting analysis of protein expression and kinase phosphorylation was preformed as described previously (Yung *et al*. 2007). In brief, placental tissue lysates were prepared using MagNA Lyser Instrument (Roche Diagnostics, Lewes, UK) with Lysing Martrix D (MP Biomedicals, Carlsbad, CA, USA) and lysis buffer containing 20 mm Tris (pH 7.5), 150 mm NaCl, 1 mm, EDTA, 1 mm EGTA, 1% Triton X‐100, 2.5 mm sodium pyrophosphate, 1 mmβ‐glycerolphosphate, 1 mm Na_3_VO_4_ and complete mini proteases inhibitor cocktail (Roche Diagnostics). Protein concentration of the tissue lysate was determined using a Bicinchoninic acid kit (Sigma‐Aldrich). Equivalent amounts of protein were resolved by SDS‐PAGE, blotted onto nitrocellulose (0.2 μm) and analyzed by enhanced chemiluminescence (Amersham Biosciences, Little Chalfont, UK) using X‐OMAT Autoradiographic film (Sigma‐Aldrich). β‐actin or Ponceau S staining was used to normalize protein loading. Films were scanned using a flat‐bed scanner (HP G4050;Hewlett Packard, Palo Alto, CA, USA) and the intensities of the bands representing phospho‐ and total kinase forms were determined from two or three different exposures (within the linear detection range) using Image J (NIH, Bethesda, MD, USA).

### Haematoxylin and eosin staining

Sections (7 μm) were dewaxed and rehydrated through xylene: twice for 5 min, and then 3 min each in the ethanol series: 100% ethanol, 90%, 70% and 50%. Slides were placed in PBS for 5 min and rinsed with water, stained in haematoxylin for 8 min, differentiated in acid‐alcohol, rinsed under tap water for 10 min, counterstained in eosin for 30 s and rinsed in water. They were rehydrated through the graded ethanol series as described above, but in reverse order, for 1 min each, placed in xylene twice for 5 min and mounted using DPX.

### Stereology

The analysis was conducted as described previously (Coan *et al*. 2004), except that the placental slides were scanned using a Nanozoomer (Hamamatsu Phonics UK, Ltd, Welwyn Garden City, UK) and analysed using newCAST software (Visiopharm Inc., Hørsholm, Denmark). In brief, six placentas from each group were randomly selected. Half of each placenta was exhaustively serially sectioned, and every 20th section was stained with haematoxylin and eosin. Placental volume was calculated using the Cavalieri technique, and the volume fraction of the different placental compartments was estimated by point counting.

### Sex determination by PCR

The sex of the pups was determined using PCR approach as described by Clapcote & Roder (2005). In brief, PCR was carried out to amplify exons 9 and 10 of *Jarid1c* and *Jarid1d* on the X and Y chromosomes, respectively, using forward primer 5'‐CTGAAGCTTTTGGCTTTGAG‐3' and reverse primer 5'‐CCACTGCCAAATTCTTTGG‐3' with the profile: 94°C for 5 min, followed by 35 cycles of 94°C for 20 s, 54°C for 1 min and 72°C for 40 s, then followed by 72°C for 10 min in a GeneTouch thermal cycler (Alpha Laboratories Ltd, Eastleigh, UK). PCR products, which are of different lengths (331 bp and 302 bp from the X and Y chromosomes, respectively), were then resolved in 2% (w/v) agarose gel and the presence of X and Y chromosomes was determined.

### Statistical analysis

All statistical analysis was performed in Prism, version 6.0 (GraphPad Software Inc., San Diego, CA, USA). Differences were tested using either the two‐tailed Student's *t* test or, when appropriate, the non‐parametric Mann–Whitney *U* test. *P* < 0.05 was considered statistically significant. For multiple comparisons, differences were tested using two‐way ANOVA, followed Bonferroni's multiple comparisons test.

## Results

### Chronic hypoxia reduces fetal weight but increases placental weight

**Figure 1:**
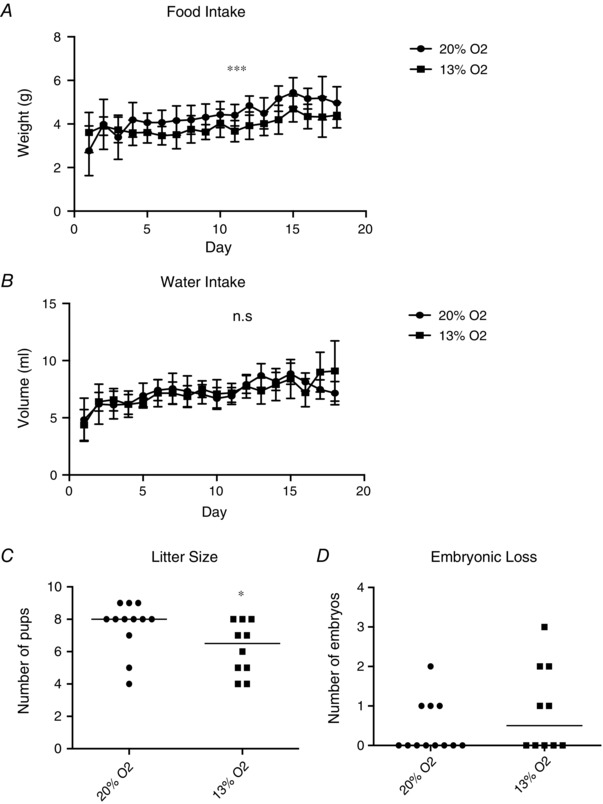
**Chronic hypoxia causes a significant reduction in food intake, but not in water intake, and reduced litter size** *A* and *B*, food and water intake were measured daily from day 0 to day 18 of pregnancy in both hypoxic and normoxic treated animals. 20% O_2_, *n* = 12; 13% O_2_, *n* = 10. *C* and *D*, the number of live and dead fetuses and resorptions was counted in every litter. *P < 0.05, ***P < 0.001; n.s, not significant.

Mice were subjected to normobaric hypoxia at 16% and 13% O_2_, equivalent to the oxygen concentration at ∼2100 m and ∼3600 m, respectively, for the whole of gestation. Food intake was reduced under 13% oxygen (*P* < 0.001), although there was no change in water intake (*P* = 0.932) at either level compared to the normoxic (20.8%) controls (Fig. 1 *A* and *B*). Litter size (live fetuses) was significantly reduced at 13% O_2_ (6.2 ± 0.5; number of litters = 10) in comparison with normoxia (7.6 ± 0.5; number of litters = 12) and 16% O_2_ (8.3 ± 0.3; number of litters = 4)(Fig. 1 *C*). There was a comparable higher incidence of embryonic loss (resorption and embryonic death) at 13% O_2_ (1.1 ± 0.4; *n* = 10) compared to normoxia (0.4 ± 0.2; *n* = 12) (Fig. 1 *D*)

**Figure 2:**
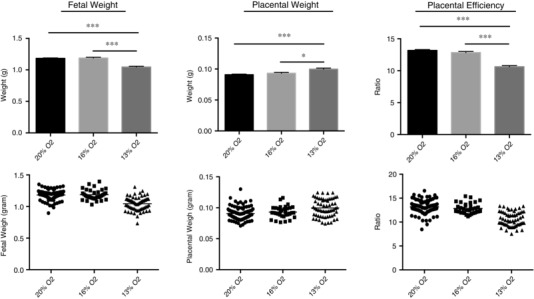
**Chronic hypoxia reduces fetal weight but increases placental weight** The presence of a copulatory plug was defined as embryonic day 0.5 (E0.5). The pregnant mice were randomly allocated to normoxic (20% O_2_) or hypoxic (16% O_2_ or 13% O_2_) groups for the entire gestation. At E18.5, animals were killed and placentas and fetuses were removed. Both placental and fetal weight were measured. Placental efficiency was calculated from fetal weight divided by placental weight. ***P < 0.001; *P < 0.05.

As expected, exposure of pregnant animals to chronic hypoxia reduced fetal growth, with a reduction of 11.7% in fetal weight at E18.5 after 13% O_2_ compared to normoxia (Fig. 2). Interestingly, placental weight was increased by 10%. No changes in fetal and placental weight were found between 16% O_2_ and normoxia (Fig. 2) and, consequently, we focused on 13% O_2_ for the remainder of the study.

### Increased maternal blood space and high growth signalling in hypoxic placentas

**Figure 3:**
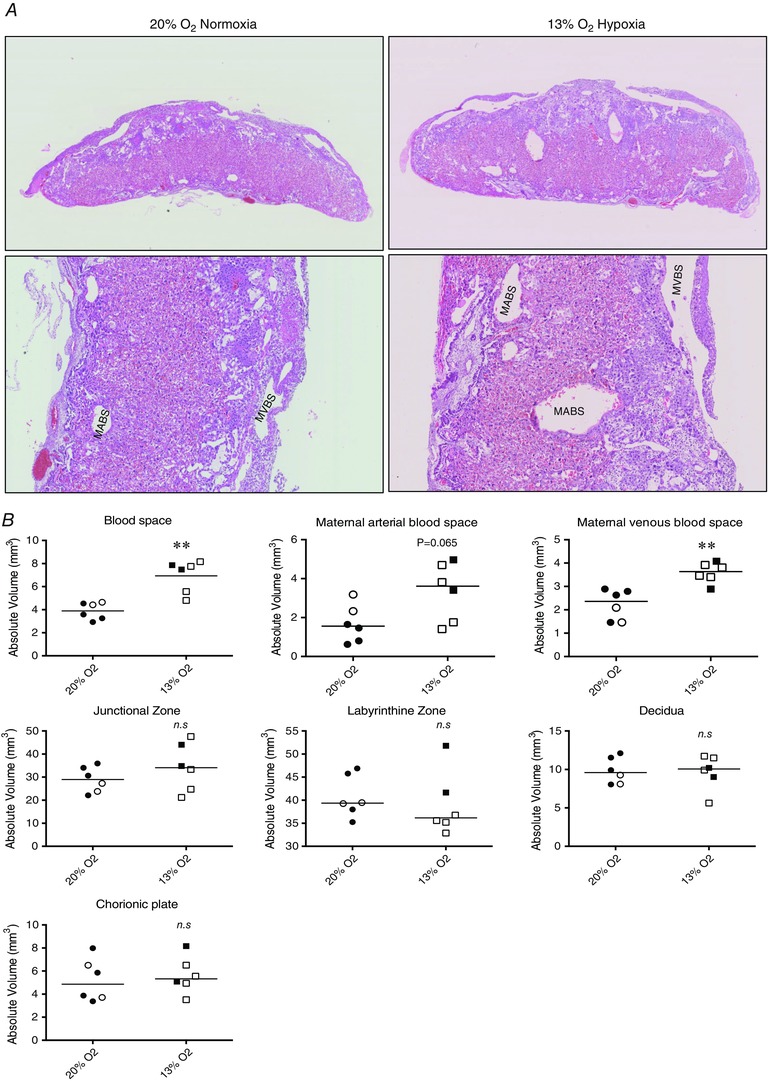
**Maternal arterial and venous blood spaces are increased in hypoxic placentas** The change in placental morphology was analysed by stereology. *A*, haemotoxylin and eosin staining showing a typical macroscopic structure of normoxic and hypoxic murine placentas under two different magnifications (1.25× for overview; 3.75× for closer view) using the Nanozoomer digital imaging system (Hamamatsu Phonics UK, Ltd). *B*, the total volume of maternal arterial blood spaces (MABS) and maternal venous blood spaces (MVBS) was increased in hypoxic placentas compared to normoxic controls. Other placental structures, including the maternal decidual layer, junctional zone, labyrinthine zone and chorionic plate, showed no change. The line in each dot‐plot graph indicates the median. Solid squares or circles represent males, whereas open squares or circles represent females. **P < 0.01; n.s, not significant.

Next, the factors that might account for the heavier placenta under 13% O_2_ were investigated. First, we examined any alteration in placental structure using a stereological approach. The isolated murine placenta can be divided into four distinctive regions based on their origin or functions, including the maternal decidua layer, junctional zone (endocrine region), labyrinthine zone (nutrient and gaseous exchange region) and chorionic plate (Georgiades *et al*. 2002). The macroscopic structure of placentas under normoxia and 13% O_2_ are shown in Fig. 3 *A*. In the hypoxic placentas, we observed large cavities in both the junctional and labyrinthine zones, which, at high magnification, were confirmed to be blood vessels because they were lined by endothelial‐like cells and contained erythrocytes. They are either maternal arterial channels supplying or venous vessels draining the placenta, depending on their location (Georgiades *et al*. 2002). Therefore, the terms ‘maternal arterial blood space’ and ‘maternal venous blood space’ were included in the stereological study.

A total of six placentas from three different litters of each group were used for stereology. No significant increase in the volumes of the major compartments of the placentas was found (Fig. 3 *B*). However, a 91% (equivalent to 3.65 mm^3^) increase in the maternal blood spaces in the 13% O_2_ placentas was observed (Fig. 3 *B*); the median maternal arterial and venous blood space volumes were increased by 132% and 54% respectively, compared to the normoxic controls. The volumes of placentas under normoxia and 13% O_2_ were 92.7 mm^3^ and 102.4 mm^3^, respectively. Hence, the 3.65 mm^3^ increase in maternal blood space cannot account fully for the 10% difference in the placental weight observed.

**Figure 4:**
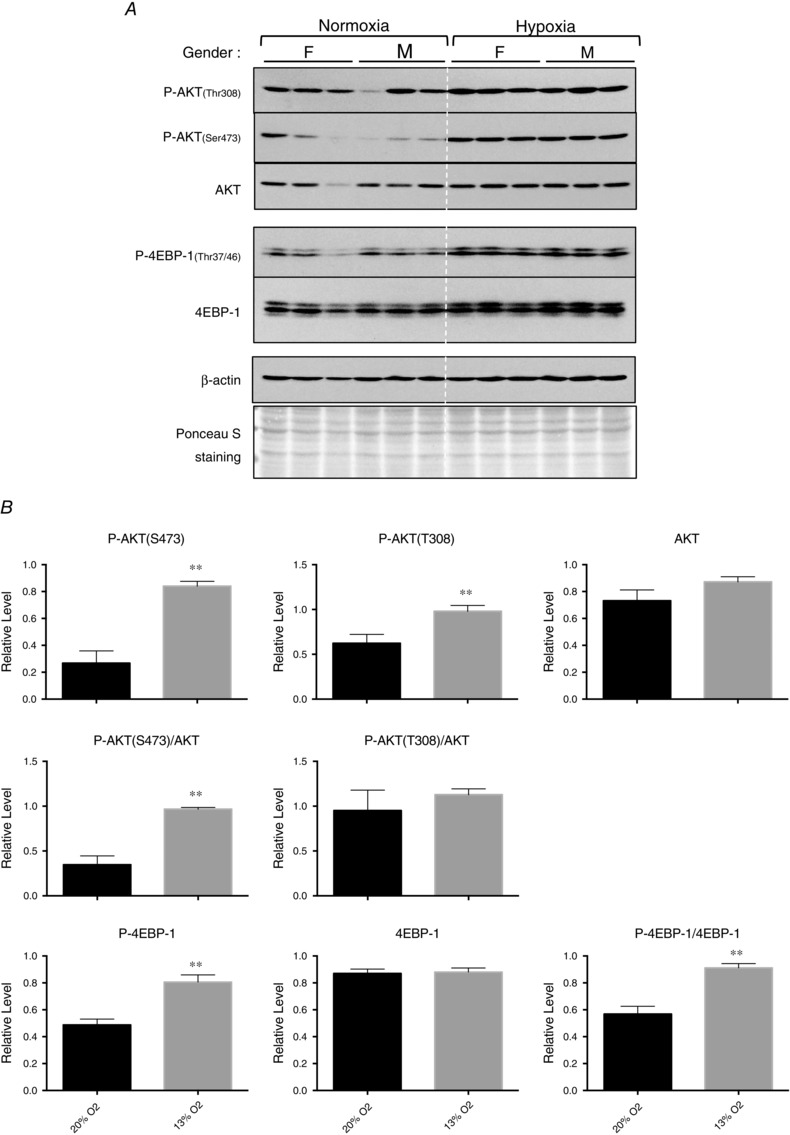
**Growth and proliferation signalling are elevated in hypoxic placentas** *A*, western blotting was used to measure the phosphorylation and total level of P‐Akt(Ser473), P‐Akt(Thr308), Akt, P‐4E‐BP1 (Thr37/46) and 4E‐BP1 using specific antibodies in both hypoxic and normoxic placentas. Both β‐actin and Ponceau S staining were used to indicate even protein loading among samples. *B*, densitometry of band intensity is expressed in arbitrary units. Phosphorylation status is presented as the ratio between phosphorylated and total protein. *P ≤ 0.05; **P ≤ 0.01.

Accordingly, we aimed to obtain evidence of enhanced placental growth under hypoxia by investigating the central Akt‐mTOR growth regulatory pathway. In comparison with normoxic placentas, there was an increase in the activity of the Akt‐mTOR pathway as shown by significant elevation of phosphorylation of Akt at both Thr308 and Ser 473, as well as of 4EBP‐1 at Thr37/46 (Fig. 4 *A* and *B*).

These results suggest that the heavier hypoxic placentas were the result of both increased placental growth and enlargement of the maternal blood vessels supplying and draining the placenta.

### Chronic hypoxia does not cause oxidative stress but does induce low‐grade ER stress

**Figure 5:**
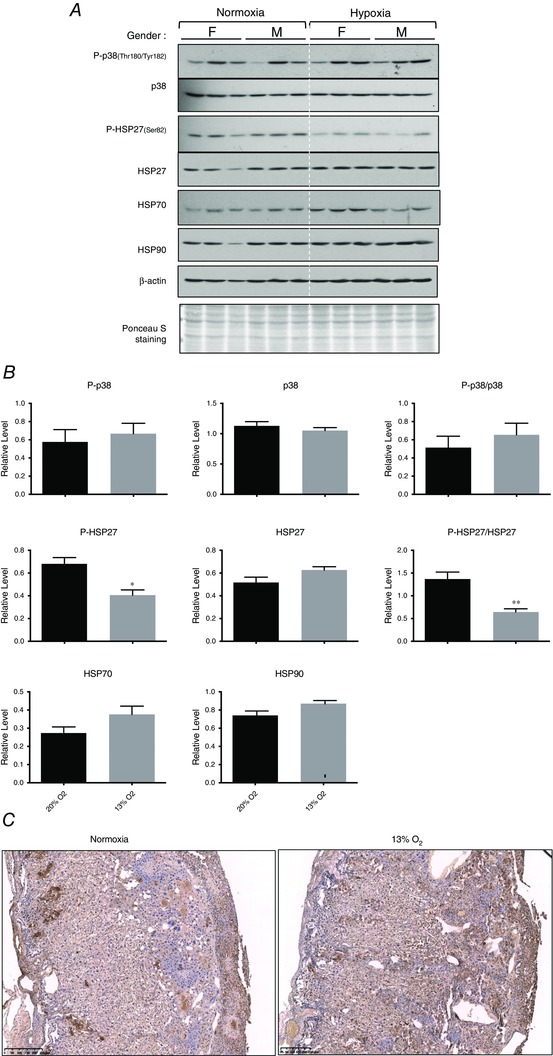
**Chronic hypoxia does not trigger oxidative stress in hypoxic placentas** *A*, western blotting was used to measure widely recognized oxidative stress markers, including P‐p38, p38, P‐HSP27, HSP27, HSP70 and HSP90, using specific antibodies in both hypoxic and normoxic placentas. Both β‐actin and Ponceau S staining were used to indicate even protein loading among samples. *B*, densitometry of band intensity is expressed in arbitrary units. Phosphorylation status is presented as the ratio between phosphorylated and total protein. Data are represented as the mean ± SEM (*n* = 6). *P ≤ 0.05; **P ≤ 0.01. *C*, immunohistochemical analysis for lipid peroxidation using an antibody specific against 4‐hydroxynonenal (4‐HNE) showed no change in immunoreactivity in any layer of the placenta. Scale bar = 250 μm.

Both oxidative and low‐grade ER stress have been reported in human placentas from high‐altitude pregnancies (Yung *et al*. 2012). Therefore, we determined whether the same stresses are also observed in the hypoxic murine placentas. Surprisingly, no increases in the major oxidative stress markers, including P‐p38 kinase, HSP70 and HSP90, were observed (Fig. 5 *A* and *B*), and phosphorylation of the small HSP27 was even reduced > 50% (Fig. 5 *B*). Furthermore, immunohistochemical staining for lipid peroxidation with antibodies directed against 4‐hydroxynonenal revealed no difference between placentas from normoxia or 13% O_2_ (Fig. 5 *C*). These results suggest that, when analysed as a pooled group, the hypoxic murine placentas did not suffer from oxidative stress.

**Figure 6:**
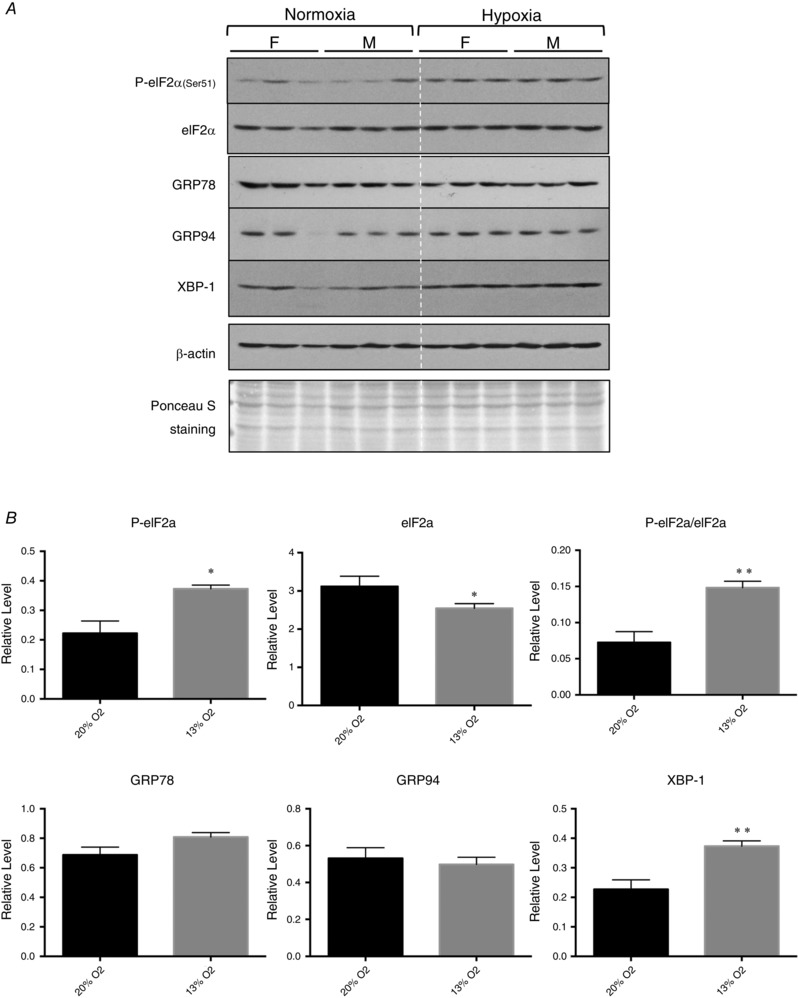
**Chronic hypoxia induces mild ER stress in hypoxic placentas** *A*, western blotting was used to measure ER stress markers P‐eIF2α, eIF2α, Grp78, Grp94 and Xbp‐1 using specific antibodies in both hypoxic and normoxic placentas. Both β‐actin and Ponceau S staining were used to indicate even protein loading among samples. *B*, densitometry of band intensity is expressed in arbitrary units. Phosphorylation status is presented as the ratio between phosphorylated and total protein. Data are represented as the mean ± SEM (*n* = 6). *P ≤ 0.05; **P ≤ 0.01.

In comparison with oxidative stress, the UPR pathways appear to be more sensitive to hypoxia (Yung *et al*. 2012). Increases of ∼100% and >70% were observed for P‐eIF2α/eIF2α and XBP‐1 respectively, whereas other markers, including GRP78 and GRP94, remained unchanged. These results indicate activation of PERK and IRE1α but not the activating transcription factor 6 pathway (Fig. 6 *A* and *B*). This indicates that hypoxic murine placentas suffer mild ER stress.

### Chronic hypoxia induces mitochondrial stress and suppresses expression of mitochondrial ETC complexes without causing intracellular energy depletion

Phosphorylation of eIF2α suppresses the translation of proteins in the mitochondrial ETC (Colleoni *et al*. 2013) and the level of some subunits was found to be reduced in human placentas at high altitude (Colleoni *et al*. 2013). Therefore, we investigated whether the high

**Figure 7:**
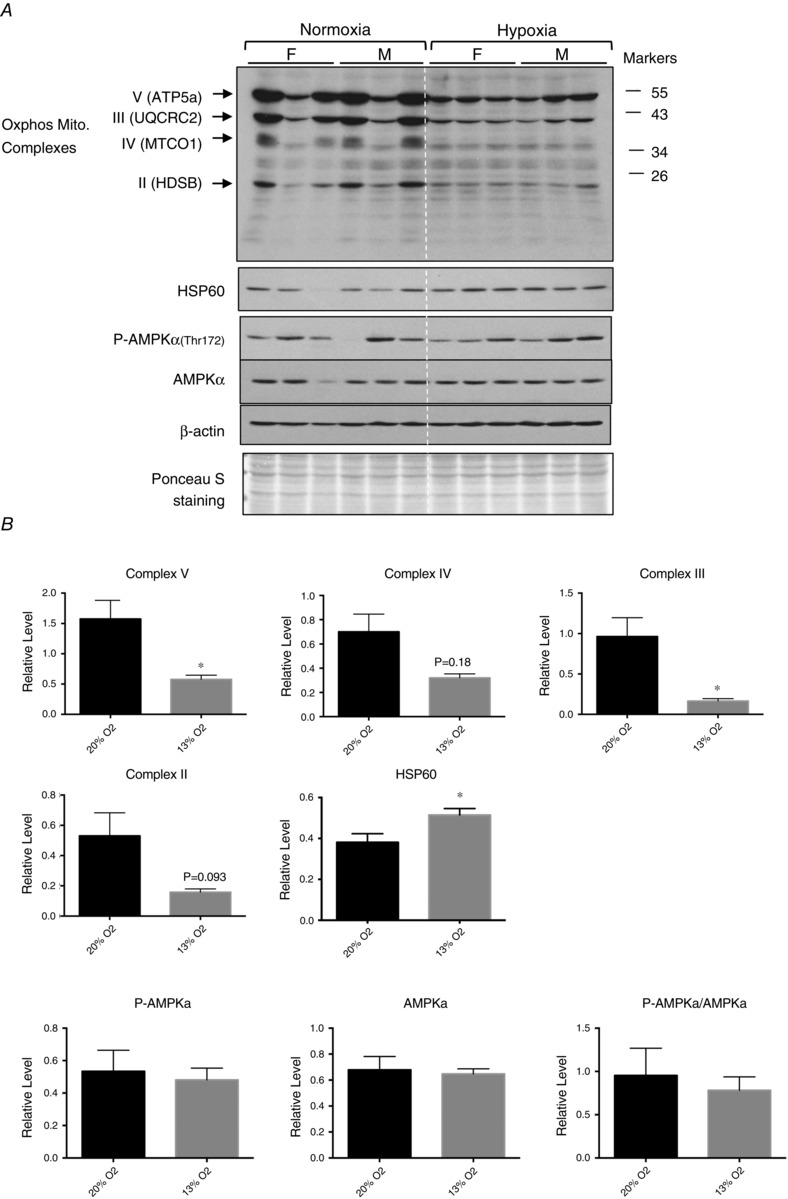
**Increased mitochondrial stress is associated with down‐regulation of mitochondrial ETC complex subunit proteins without causing intracellular energy depletion in hypoxic placentas** *A*, western blotting was used to measure the mitochondrial stress marker, HSP60; a number of mitochondrial ETC complexes subunits; and the energy sensing kinase, P‐AMPKα and AMPKα, using specific antibodies in both hypoxic and normoxic placentas. Both β‐actin and Ponceau S staining were used to indicate even protein loading among samples. *B*, densitometry of band intensity is expressed in arbitrary units. Phosphorylation status is presented as the ratio between phosphorylated and total protein. Data are represented as the mean ± SEM (*n* = 6). *P ≤ 0.05.

**Figure 8:**
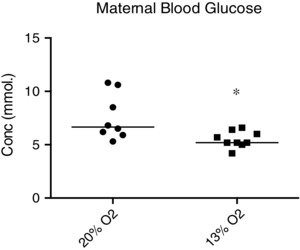
**A reduction in maternal blood glucose in hypoxic animals** All animals were killed by neck dislocation to minimize stress‐induced glucose elevation. Blood glucose was measured immediately in tail blood using a glucometer. 20% O_2_, *n* = 8; 13% O_2_, *n* = 9. The line indicates the median value of each dataset. *P < 0.05.

P‐eIF2α in the hypoxic murine placentas was associated with changes in the mitochondrial ETC. HSP60 is a mitochondrial chaperone responsible for the transport and refolding of proteins from the cytoplasm into the mitochondrial matrix. It was increased significantly by 35% under hypoxia (Fig. 7 *A* and *B*), suggesting the probable existence of mitochondrial stress. Indeed, the level of some mitochondrial ETC complex subunits was reduced by >60%, including complex V (ATP5a), complex IV (MTCO1), complex III (UQCRC 2) and complex II (SDHB) (Fig. 7 *A* and *B*). Surprisingly, despite these changes, there was no indication of an energy crisis in the hypoxic placentas because phosphorylation of the energy sensing kinase, AMPKα, showed no difference. However, we did observe that the maternal glucose concentration at E18.5 was reduced significantly by 29% [7.6 ± 0.8 mmol (*n* = 8) to 5.5 ± 0.3 mmol (*n* = 9) *P* = 0.011] (Fig. 8). These results suggest that hypoxic placentas might carry out more anaerobic respiration to compensate for any reduction of ATP generation resulting from the defective mitochondrial ETC.

### Sex‐specific outcomes on placental growth in response to chronic hypoxia

**Figure 9:**
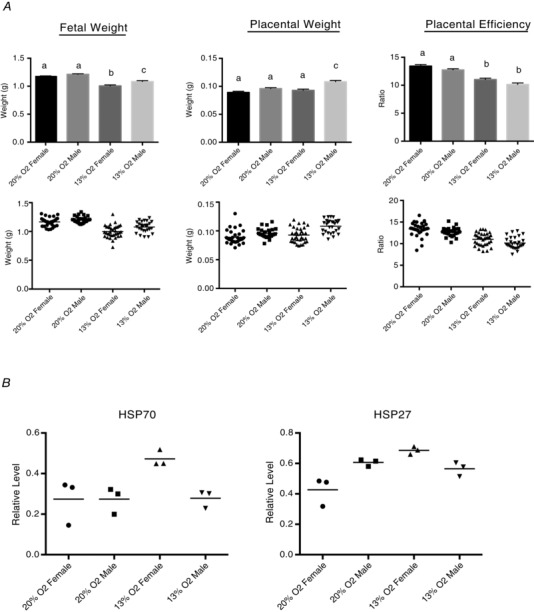
**Sex‐specific outcomes on placental growth in response to chronic hypoxia** *A*, placental and fetal weights in Fig. 2 were reanalysed after taking the sex of the fetus into account. The new data indicated that hypoxic male placentas were heavier than female ones, whereas male fetuses were less growth restricted than females. *B*, reanalysis of all the western blot data revealed that only female placentas suffered hypoxia‐induced oxidative stress as indicated by elevated HSP70 and HSP27 proteins. The line in each dot‐plot graph indicates the mean value (*n* = 3). Different letters indicate groups that are significantly different using two‐way ANOVA, followed by Bonferroni's multiple comparisons test. *P ≤ 0.05; **P ≤ 0.01.

There is growing evidence that sexual dimorphism plays a crucial role in determining the outcome of pregnancies affected by stress (Gabory *et al*. 2013). Therefore, we reanalyzed the data on fetal and placental weights using two‐way ANOVA to take the sex of the offspring into account. In the normoxic group, there were 27 male and 32 female pups and, in the hypoxic group, there were 30 male and 34 female pups (Table 1). Both oxygen concentration and sex of the offspring had significant effects on placental and fetal weights, as well as placental efficiency (*P* < 0.005 in all cases). There was a significant interaction between the two effects for placental weight (*P* < 0.05) but not for the other parameters. Placental weight was greatest in males under hypoxic conditions (Fig. 9 *A*). These effects could not be explained by differences in litter size because this was constant (*P* > 0.05) across the groups (Table 1). Placental efficiency, as expressed by the ratio between fetal and placental weight, did not show a significant difference between females and males under hypoxia, suggesting that the heavier placentas in males mitigated the growth restriction induced by hypoxia.

**Table 1:** Distribution of sex and fetal condition in each litter

	Live pups	Female	Male	Embryonic death	Litter size
20% O_2_					
1	8	2	6	0	8
2	8	5	3	0	8
3	9	7	2	1	10
4	7	5	2	0	7
5	8	6	2	0	8
6	9	4	5	1	10
7	8	4	4	0	8
8	4	1	3	2	6
9	5	3	2	0	5
10	9	ND	ND	0	9
11	8	ND	ND	0	8
12	8	ND	ND	1	9
Total	91	–	–	5	96
Mean	7.6	4.1	3.2	0.4	8
13% O_2_					
1	7	5	2	2	9
2	5	1	4	0	5
3	8	4	4	0	8
4	6	4	2	0	6
5	8	4	4	1	9
6	5	2	3	2	7
7	4	2	2	3	7
8	8	5	3	0	8
9	4	2	2	1	5
10	7	5	2	0	7
Total	62	–	–	9	71
Mean	6.2	3.4	2.8	0.9	7.1

ND, not determined.

Additionally, we re‐examined the levels of total protein and, where appropriate, phosphorylation of various markers of oxidative and ER stress, the mitochondrial ETC, and kinases in the AMPK and Akt‐mTOR pathways. Most of the markers did not show a difference between the sexes, although HSP70 and HSP27 appeared to be consistently increased in females under hypoxia, indicating potential exposure to oxidative stress (Fig. 9 *B*). These results suggest that female placentas may be more susceptible to hypoxia‐induced stresses than male placentas, possibly rendering them less able to respond effectively and resulting in more severe fetal growth restriction. Larger studies enabling statistical analyses are required to confirm this point.

## Discussion

Normobaric housing of pregnant mice at 13% oxygen for their entire gestation induced ∼12% reduction in fetal weight. This is greater than the 7% reduction reported for mice housed under 12% from E14.5 onwards (Cuffe *et al*. 2014). Interestingly, the detrimental effect on fetal growth induced was sex‐specific, with female fetuses being affected to a greater extent than male fetuses. The birth weight of human males is known to be heavier than that of females (Misra *et al*. 2009), and this effect is magnified by altitude. In a study of pregnancies of European ancestry, male birth weight was ∼1.8% heavier than female birth weight at low altitude (430 m), whereas this difference widened to ∼3.7% at high altitude (3600 m) (Soria *et al*. 2013). This finding suggests the involvement of sex differences in hypoxia‐induced growth restriction, which is consistent with our observations.

By contrast, placental weight was increased. This change was probably not induced by the small drop in food intake because we have previously reported a reduction in placental weight in undernourished mice (Coan *et al*. 2010). More probably, it is a result of the chronic hypoxia. Cuffe *et al*. (2014) reported no change in placental weight in mice after exposure during mid‐ to late‐gestation, although their data indicate a similar trend to a reduction in placental efficiency. In rats, placental weight was significantly increased after exposure to 13% oxygen from day 6 (Richter *et al*. 2012), whereas a greater placental cross‐sectional area was reported under 11% oxygen (Rosario *et al*. 2008). Further analysis indicated that sex‐specific changes in the placenta may account for the male–female differences in the fetal weight that we observed. The placentas of male fetuses were heavier, and demonstrated less evidence of oxidative stress than their female counterparts, and so may have been able to compensate for the reduced maternal *P*_a_O_2_. Nonetheless, placental efficiency did not show a significant difference between male and females. Maintaining growth under adverse conditions may be a risky approach by the male fetus that could result in higher embryonic lethality.

Oxygen crosses the placenta by simple diffusion, and the principal driving force is the concentration gradient between the two circulations. A maternal–fetal gradient of ∼10 mmHg is considered to be necessary for placental exchange (Browne *et al*. 2015) and may have influenced the evolution of the placental mammals (Falkowski *et al*.

2005). Oxygen crosses cell membranes freely, and so placental exchange is considered to be flow‐limited rather than diffusion‐limited (Wilkening & Meschia, 1983). A high blood flow on either side of the placenta will refresh and deplete the supply and recipient pools, respectively, ensuring that the maximum gradient is maintained. This effect will become even more important in the high‐altitude situation where the gradient is inevitably lower as a result of reduced maternal *P*_a_O_2_. Indigenous populations demonstrate higher uteroplacental blood flows at high altitude than recent migrants, and this is assumed to explain their higher birth weights (Browne *et al*. 2015). Hence, the enlarged maternal arterial blood spaces supplying (and venous spaces draining) the placenta seen in our hypoxic animals may represent a compensatory mechanism. They are consistent with the concept that structural changes are more successful on the maternal rather than the fetal side of the high‐altitude placenta (Mayhew, 1991). Trophoblast invasion and remodelling of the uterine spiral arteries may mediate this effect because these are enhanced in rats maintained under hypoxia during pregnancy, leading to greater vessel diameters (Rosario *et al*. 2008). The critical period for exposure was found to be E8.5–9.5, and so this effect will not have operated in the study of Cuffe *et al*. (2014), which only involved exposure to hypoxia during mid‐ to late‐gestation. This could account for the fact that no increase in placental weight was observed in their study.

The changes in maternal blood volume could not fully account for the increase in placental weight. Activation of Akt‐mTOR signalling suggests that there may also have been enhanced placental growth under hypoxia because this pathway plays a crucial role in the regulation of placental size. Akt‐mTOR signalling is down‐regulated in placentas from growth restricted pregnancies (Yung *et al*. 2008) and elevated in pregnancies of obese women with large babies (Jansson *et al*. 2013). Therefore, the increased Akt‐mTOR signalling in the hypoxic placentas is consistent with our finding of a heavier placenta, although the difference in volume generated may have been too small to be detected stereologically because of the small sample size. This result was contradictory to our previous finding of down‐regulation of Akt‐mTOR signalling in human placentas from high altitude, although, in that study, there was a concomitant reduction in growth of the placental villous tree (Yung *et al*. 2012). Activation of Akt signalling by hypoxia has been reported in other cell types (Alvarez‐Tejado *et al*. 2001), although the mechanisms that are responsible remain unclear. Activation of *de novo* synthesis of RNAs and/or proteins is required because the global protein and RNA synthesis inhibitors, cyclohexamide and actinomycin D, both suppressed hypoxia‐induced Akt phosphorylation (Alvarez‐Tejado *et al*. 2001). Sex differences in the response of the human placenta to adverse stimuli are increasingly being recognized. Differences in gene expression associated with high‐level functions, such as protein synthesis, hormone secretion and growth, have been reported (Clifton, 2010; Osei‐Kumah *et al*. 2011; Buckberry *et al*. 2014). Further analyses are required to determine whether they might account for the differences observed in the present study.

In addition, our findings indicate that female, but not male, placentas might suffer from low‐grade oxidative stress under hypoxia because they express higher levels of HSP70 and HSP27. Unlike the stress kinase p38, which reacts rapidly in response to stress, HSPs are usually the second wave for long‐term protection against stress‐induced denaturation of proteins (Feder & Hofmann, 1999). The mechanisms underlying the greater susceptibility of a female placenta to hypoxia‐induced oxidative stress are unknown. However, we speculate that they may involve sex‐specific differences in placental cytokine profile. Cytokines such as tumour necrosis factor (TNF)α can induce the production of reactive oxygen species, resulting in oxidative stress. In pregnancies complicated by asthma, the female placenta expresses high levels of mRNAs encoding pro‐inflammatory cytokines, including TNFα, interleukin (IL)‐1β, IL‐6, IL‐5 and IL‐8, whereas there is no significant change in the male placenta compared to corresponding controls (Scott *et al*. 2009). Additionally, maternal concentrations of TNFα, IL‐6 and IL‐8 are increased significantly during the third trimester of pregnancy at high altitude (Coussons‐Read *et al*. 2002), indicating that hypoxia potentially promotes placental cytokine production. Furthermore, analysis of sexual dimorphism in zebrafish liver enzymes reported higher transcription of the gene for glutathione peroxidase 1 in male compared to female organs (Zheng *et al*. 2013). Therefore, the high pro‐oxidant production with low anti‐oxidant defence is consistent with the oxidative stress observed in female placentas.

Although there were differences in the oxidative stress detected in the male and female hypoxic placentas, the degree of ER stress was equivalent. Activation of the different UPR pathways is closely linked to the severity of ER stress (Yung *et al*. 2008). In the human placenta at high altitude, we only detected activation of PERK/eIF2α arm, whereas, in the hypoxic murine placentas, IRE1/XBP‐1 signalling was also increased, indicating a potentially higher degree of ER stress. However, hypoxia can induce different severities of ER stress in different placental cell types (Yung *et al*. 2012) and so these data should be interpreted with caution. ER stress and activation of the UPR are usually related to protein synthesis inhibition through phosphorylation of eIF2α, although this inhibition is selective. mRNAs containing small upstream open reading frames within their 5′‐untranslated regions or internal ribosome entry sites sequences are translated independent of eIF2α regulation (Lu *et al*. 2004). Translation initiation is also regulated by eukaryotic initiation factor 4 (eIF4) family members (Gingras *et al*. 1999), which in turn are regulated by 4EBP‐1. 4EBP‐1 binds to eIF4E, preventing its interaction with eIF4 family members during the formation of the translation initiation complex. Phosphorylation of 4EBP‐1, which is mediated by mTORC1, blocks this interaction (Mothe‐Satney *et al*. 2000). Therefore, the increased mTOR activity in hypoxic murine placentas indicates that eIF2α independent protein translation is probably ongoing, thereby increasing placental growth.

Consistent with an arrest of translation, we did observe a remarkable reduction in protein subunits of the mitochondrial ETC complexes. We have previously reported that increased phosphorylation of eIF2α induced by salubrinal, a specific phosphatase inhibitor for p‐eIF2α, suppresses ETC complex subunits (Colleoni *et al*. 2013). Additionally, we also demonstrated down‐regulation of the subunits in high‐altitude placentas (Colleoni *et al*. 2013). Reduction of ETC complex II activity probably reduced the number of electrons feeding into the ETC, thereby preventing electron leakage and production of reactive oxygen species. This effect could help to explain why no change in oxidative stress was observed in the male placentas, and only low‐grade stress in the female ones. Despite the reduction in ETC complexes, activity of the energy sensing kinase, AMPKα, did not change, indicating no crisis in cellular energy level. However, we did observe a reduction of maternal glucose concentration in the hypoxic animals, indicating that there may have been a general shift to anaerobic respiration as the major energy source.

Taken together, our results show that the murine placenta undergoes fine‐tuning mediated by growth signalling and stress‐response pathways to ensure optimal fetal survival and growth in response to chronic hypoxia. The female appears to follow a conservative approach, generating a smaller placenta and fetus to ensure survival of the offspring, whereas the male appears to be more aggressive with increased placental growth to extract more resources from the mother and maintain maximal fetal growth. The latter is a more risky strategy because there may be more fetal and neonatal deaths, although it is consistent with what is reported in humans (Eriksson *et al*. 2010). This sexual dimorphism in placental responses towards adverse conditions may be beneficial with respect to the continuity of the species by natural selection because small populations of males are sufficient to mate with larger numbers of females.

## Additional information

### Competing interests

The authors declare that they have no competing interests.

### Author contributions

GJB, DSC‐J and HWY designed the study. HM, JHWV and HWY performed the experiments. GJB, DSC‐J and HWY analysed the data and wrote the paper. All authors approved the final version.

### Funding

This study was supported by a grant from the Wellcome Trust (084804/2/08/Z).
